# Experiences of parents with an adolescent abusing substances admitted to a mental health institution in Giyani, South Africa

**DOI:** 10.4102/curationis.v43i1.2139

**Published:** 2020-11-05

**Authors:** Evelyn N. Hlungwani, Nompumelelo Ntshingila, Marie Poggenpoel, Chris P.H. Myburgh

**Affiliations:** 1 Department of Nursing Sciences, Faculty of Health Sciences, University of Johannesburg, Johannesburg, South Africa; 2 Department of Educational Psychology, Faculty of Education, University of Johannesburg, Johannesburg, South Africa

**Keywords:** adolescent, experiences, parents, qualitative, substance abuse

## Abstract

**Background:**

Substance abuse by adolescents may be a problem that contributes to their mental illness. Substance abuse does affect not only the individual who is abusing it but also friends, family and the whole community. The adolescent abusing substances may be mentally unstable and have unpredictable behaviour. There is no research on the experiences of parents with adolescents abusing substances in Giyani, South Africa.

**Objective:**

The purpose of the study was to explore and describe the experiences of parents with adolescents abusing substances admitted to a mental health institution in Giyani.

**Method:**

A qualitative, exploratory, descriptive and contextual research design was used. Data were collected by means of conducting individual, in-depth, phenomenological interviews, observations and field notes. The following central question was asked to the participants: ‘How is it for you to have an adolescent who is abusing substances’. Data were analysed by using a thematic method of coding. An independent coder analysed data together with the researcher, and consensus was reached.

**Results:**

Four themes emerged from the data: parents experienced uncontrolled thoughts regarding their adolescent abusing substances, not being able to control their adolescent abusing substances through discipline, negative feelings regarding their adolescent abusing substances and negative consequences regarding their adolescents abusing substances.

**Conclusion:**

From the study result, it is clear that parents with adolescent abusing substances need professional assistance and support as evidenced by the challenges faced in terms of promoting, maintaining and restoring their mental health. Psychiatric nurses should take responsibility to educate the community about substance abuse, for example offering school health programmes. Further research studies can also be conducted in other villages to gain a greater understanding of those parents’ experiences with an adolescent abusing substances.

## Introduction and overview

Substance abuse is a significant problem contributing to individuals’ mental illness. It does affect not only the individuals who are abusing substances but also their family, friends, community and the healthcare system. Substance abuse occurs in all age groups, but adolescents are the most vulnerable population (Taylor 2018:17).

According to the World Health Organization (WHO 2014:15), the percentage of adolescents abusing substances is increasing in all communities, irrespective of the support that parents provide and what other stakeholders are doing to reduce this social problem. Ondieki and Makua (2012:506–513) are of the opinion that the issue of drug abuse has been in existence for thousands of years; it has been an integral part in most societies. Currently, substance abuse is a problem experienced by both young and old, although its impact tends to be particularly intense amongst adolescents.

In South Africa, 12% of youth experiment with alcohol before the age of 13 years (Reddy et al. 2010:16). There is also a countrywide increase in substance intake amongst adolescents, including the Limpopo Province. The Limpopo Department of Social Development (2013:51) stated that 26.8% of adolescents between the ages of 11 and 20 years abuse substances. The South African Community Epidemiology Network on Drug Use (SACENDU 2017:22) reported that there has been an increase of substance abuse in the northern regions from 22% in 2014 to 26% in 2016. This is a major cause of concern as it increases the risk of human immunodeficiency virus (HIV) infection, teenage pregnancy, school dropout, delinquent or criminal behaviour, and mental illness. The peer group is significantly linked with alcohol misuse during this stage of adolescence (Furguson & Meehan 2011:6–12). Parents also play a vital role during adolescence as young adolescents seek guidance from their own parents’ habits.

There are many types of substances being abused. These are classified as depressants, stimulants, opioids, hallucinogens and cannabinoids (Nitescu 2019:245). People abusing substances show unpredictable behaviour, and they have physical and emotional problems such as depression, aggression and violence. Many substances cause forms of intoxication, which alter the judgement and perception of the person. Adolescents start behaving strangely and are seen to be irritable and aggressive (Ali et al. 2011:25). Adolescents abusing substances experience feelings of having more energy. This is often perceived to improve their performance at manual or intellectual tasks. They may be extremely happy or exuberant, with grandiose ideas, and spend excessive sums of money (Ali et al. 2011:25). Adolescents abusing substances do not care about their physical appearance and often fail to attend to their personal hygiene.

Dauber et al. (2018:67) state that not all adverse consequences of substance abuse are delayed. For instance, female adolescents turn to prostitution to support their expensive substance abuse habit. Some adolescents commit suicide or murder, sometimes caused by substance-induced psychosis. Treatment for adolescents who abuse substances and who have psychotic symptoms should thus be integrated and include management of both the psychosis and the addiction (Dauber et al. 2018:67).

Parents experience stress and stigma attached to their adolescent who abuses substances. There also seems to be a link between substance abuse and mental illness, as many adolescents suffer from mental illness because of substance abuse (Kalam & Mthembu 2018:468). Parents become angry and disappointed with their adolescents abusing substances, as it destroys the adolescent’s future and the expectations they may have for their child are not met (Smith, Estefan & Caine 2018:517). Parents may experience stress in looking after adolescents who abuse substance as most of these abusers end up being affected by substance-related mental disorders, which may be experienced as a ‘family burden’. The majority of problems surrounding adolescent substance abuse have an impact on parents, yet little is known about their experiences (Smith et al. 2018:512).

Substance abuse poses a far greater threat to adolescents than many parents care to admit (Taylor 2018:14). Childhood is a period of significant growth and change. Adolescents tend to be uncertain about themselves and parents need to be prepared to talk frankly with their children about alcohol from a very early age, such as when they are in elementary school. Taylor (2018:15) states that adolescence is the most common period for the first experience with substances. Although adolescents who use psychoactive substances tend to progress from nicotine to alcohol to marijuana and then to drugs that are perceived to be more dangerous, substance use patterns seem to be related to availability (Stuart 2013:547).

The author who initially conducted the research (Hlungwani 2020:6) on which this article is based realised that most parents who visit their children in the hospital are complaining that their adolescents steal their valuables from home and sell them for drugs (Hlungwani 2020:55,58,62; Calder 2012:144; Masombuka 2013:8; Waini 2015:9). The valuables include portable equipment such as TVs, cell phones, microwaves and money. Some parents stated that these adolescents prepare themselves and leave for school, only for the parents to discover that they are not attending classes (Hlungwani 2020:59, 61). Other authors (Bradford Health Services 2020:np; Choate 2015:462; Dreyer 2012:3; Hoeck & Van Hal 2012:9; Lebese, Ramakwela & Maputle 2014:338) also supported the parents’ statement that adolescents do not attend school.

Based on the above studies, it is evident that studies have been conducted concerning substance abuse amongst adolescents across the world, including South Africa. Giyani in the Limpopo Province is a deprived rural part of the country, where such studies have not been conducted before. The reason why the researchers decided to conduct this study in Giyani was the unique cultures, values and traditions. There is a gap in the description of the experiences of parents who have adolescents abusing substances in Giyani. This article thus explored and described the experiences of parents with adolescents abusing substances as they provide care for them. The research question that arose from this problem statement is as follows:

- What are parents’ experiences of an adolescent abusing substances admitted to a mental health institution in Giyani?

### Research purpose

The purpose of this article was to explore and describe the experiences of parents with an adolescent abusing substances admitted to a mental health institution in Giyani.

## Definition of key concepts

### Experiences

Experience is the practical involvement in an activity or event, which affects a person directly (Hole & Hawker 2015:198). A phenomenological study describes the common meaning for individuals of their experiences of a concept or phenomenon (Creswell 2014:76). In this article, experiences refer to the thoughts, feelings, values, beliefs and attitudes of parents with an adolescent abusing substances, admitted to a mental health institution in Giyani.

### Parents

Parents are two or more people who regard themselves as family and who perform some typical family functions. These people may or may not be related by blood or marriage, and may or may not live together (Turnbull & Turnbull 2015:14). In this discussion, parents are people who raise adolescents and take care of them.

### Adolescent

Adolescence is the period of life when a child develops into an adult; it is the period from puberty to maturity terminating legally at the age of majority (the age at which full civil rights are accorded) (Merriam-Webster Dictionary 2012:368). In this article, adolescents refer to children between the ages of 15 and 17 years.

### Substance

‘Substance’ means chemical, psychoactive substances that are prone to be abused, including tobacco, alcohol, over-the-counter drugs, prescription drugs and substances defined in the *Drugs and Drugs Trafficking Act* (No 140 of 1992). In this article, substances are illicit drugs that are consumed by adolescents. A substance is also referred to as a drug at grassroots level in the community.

### Substance abuse

Substance abuse is when the person shows maladaptive substance use resulting in a clinically significant failure to fulfil major role obligations at work, school or home (eds. Uys & Middleton 2014:576). Recurrent substance-related behaviours include arrests for substance-related misconduct or possession of illegal substances. In this article, substance abuse refers to the sustained use of illicit drugs by the adolescents.

## Research design and method

### Research design

The research design is qualitative, exploratory, descriptive and contextual. This design was applied to ascertain the fundamental nature of the experiences of parents with an adolescent abusing substances (Creswell 2014:56–57). This design was considered appropriate because it focusses on gaining insights by means of discovering meaning about the life experiences of parents with an adolescent abusing substances.

### Research setting

The research was conducted in Giyani, which is one of the municipalities falling within Mopani District Municipality in the Limpopo Province (Greater Giyani Municipality 2019:1). In this study, Giyani refers to one of the cities in Limpopo Province, South Africa, where the mental health institution that constitutes the research setting is situated. This mental health institution has 400 beds of which 354 were in use. The mental health institution admits mental healthcare users with various mental health challenges, as well as adolescents diagnosed with substance abuse. The setting for this study was the homes of the parents with an adolescent abusing substances admitted to a mental health institution in Giyani.

### Research method

#### Population and sample

The population included the parents of adolescents abusing substances admitted to a mental health institution in Giyani. A purposive sampling method was used to select the participants. The author who initially conducted the research selected the participants to be included in the study, namely the parents of an adolescent abusing substances. The sampling size was determined by the in-depth information required for the author to gain insight into the phenomenon (Gray, Grove & Sunderland 2017:347). The sample size was determined by data saturation when additional sampling did not provide new information. The participants were recruited through the mental health institution where their adolescents were admitted. The participants were contacted to invite them to take part in the study. If the parents decided to participate in the study, the researcher contacted the interested parents to interview them. The inclusion criteria were as follows:

- Parents who have adolescents between the ages of 15 and 17 years who are abusing substances.
- Parents who have given consent to participate in the study.
- Parents who are either male or female.
- Parents who are able to communicate in English, Xitsonga or Sesotho.

#### Data collection

In-depth, phenomenological interviews, observation and field notes are the data collection methods that were used until data saturation was reached. Saturation of data occurs when additional sampling provides no new information, only redundancy of previously collected data (Gray et al. 2017:129). Data saturation was reached during the fifth interview when the same themes emerged repeatedly. Three extra interviews were conducted to confirm the existing themes.

With the qualitative methodology, data collection and analysis occur at the same time (Creswell 2014:187). Data were collected by using in-depth, phenomenological interviews to explore and describe the phenomenon. The researcher also employed bracketing to avoid bias in the study (Gray et al. 2017:156). The central question posed to the participants was as follows: *How is it for you to have an adolescent who is abusing substances?*

Each interview with the parents of adolescent abusing substances lasted for 45–60 min. The interviews were conducted in a conducive environment free from distractions and noise, and the interviews were audio-recorded with permission from the participants. Observations and field notes were made as part of the data collection methods. The researcher’s observations were retained by using field notes to record activities as well as the researcher’s interpretations of those activities (LoBiondo-Wood & Haber 2014:276). The researcher also interpreted the observations made, which assisted the researcher in making sense of what the participants mentioned. Parents were initially reluctant to express their feelings of being unwanted, unloved and rejected by the community.

#### Data analysis

The transcribed interviews and field notes collected during the interviews on the experiences of parents with an adolescent abusing substances admitted to a mental health institution in Giyani were reduced and organised to give meaning to the data. An independent coder, experienced in qualitative research, was utilised to analyse the data separately from the researcher. The researcher and the independent coder met for a consensus discussion on the results of the data analysis. The researcher used the thematic coding method (Creswell 2014:368) to analyse and make sense of data that were collected. To analyse qualitative data, the researcher sought meaning from all the raw data that were available. The results were recontextualised within the literature.

### Measures to ensure trustworthiness

Guba’s model (Lincoln & Guba 2005:290) of trustworthiness was adhered to. Four important aspects when dealing with trustworthiness in the qualitative paradigm, namely credibility, transferability, dependability and confirmability, were applied throughout the study. Credibility was improved by prolonged engagement in the data collection process and enhanced triangulation. The researcher used in-depth phenomenological interviews, observation and field notes to ensure the credibility of the study. Member checking was also achieved when the researcher validated and clarified her interpretations with the participants.

Transferability was enhanced by providing a dense description of the results with direct quotations from the participants’ interviews. A clear and detailed demographic description of the selected samples was provided to transfer the findings to similar contexts. The researcher also triangulated multiple sources of data.

Dependability was enhanced by means of a dense description of the research methodology, where all aspects of the research were fully described. The researcher conducted in-depth, phenomenological, individual interviews and wrote field notes, and she ensured the audit trail by audio recording the interviews. The interviews and field notes were then coded and recoded.

Confirmability was applied through an audit trail of the methods, data and analytical processes presented as a decision trail, subjected to an external audit by a reviewer introduced towards the end of the study. The transcripts, audio-records and field notes will be kept for 2 years as proof of the chain of evidence.

### Ethical consideration

The researcher obtained approval from the ethics committees of the University of Johannesburg, Faculty of Health Sciences Research Ethics Committee (Rec-241112-035) and Limpopo Department of Health Research Ethics Committee (Ref: LP2018), as well as the management of the institution (Ref: S4/2/2), where she worked before the study commenced. The researcher contacted the parents to arrange interviews with them in their home settings. The following four ethical principles governed the study: autonomy, non-maleficence, beneficence and justice (Dhai & McQuoid-Mason 2011:14–15).

The principle of autonomy was respected by treating participants as autonomous agents who have the freedom to conduct their lives as they choose without external control. The participants were allowed to make an informed decision before signing an informed consent form. Moreover, their confidentiality was respected throughout the study by the researcher not exposing their names.

The principle of non-maleficence was adhered to by conducting interviews in a private place to promote the comfort and confidentiality of the participants. The risk–benefits ratio of the participants was assessed prior to their involvement in this study. Participants were referred to a social worker for debriefing sessions should they have needed it after the interview.

To ensure the principle beneficence, the researcher was sensitive to any signs of emotional discomfort and scheduled individual emotional support as needed. The researcher asked appropriate questions to avoid discomfort that could have occurred throughout the interviews.

To ensure the principle of justice, the researcher guaranteed the fair selection of the participants in the study. The researcher excluded social, cultural, racial and sexual biases in the society (Gray et al. 2017:106) and selected the participants based on research requirements and not the vulnerability or compromised position of certain people (Polit & Beck 2017:156). The participants were treated fairly and with respect.

## Findings

### Participants’ demographics

Eight parents with adolescents abusing substances admitted to a mental health institution in Giyani participated in the primary study. The parents were between the ages of 42–52. They were all black South Africans and met the sampling criteria. The in-depth, phenomenological interviews, which lasted for 45–60 min each, were conducted in Tsonga, and direct quotations were translated into English (Hlungwani 2020:47–48).

### Findings of the in-depth interviews

The analysis findings of the transcribed in-depth interviews are summarised in Table 1.

**TABLE 1:** Themes.

Themes	Description
Theme 1	Parents experienced uncontrolled thoughts regarding their adolescent abusing substances.
Theme 2	Parents experienced not being able to control their adolescent abusing substances through discipline.
Theme 3	Parents experienced negative feelings regarding their adolescent abusing substances.
Theme 4	Parents experienced negative consequences regarding their adolescent abusing substance.

*Source*: Hlungwani, E.N., 2020, *Experiences of parents with an adolescent abusing substances admitted to a mental health institution in Giyani*, MNSC Psychiatry and Mental Health Nursing, University of Johannesburg, Johannesburg

The themes are discussed based on those presented in Table 1 (Hlungwani 2020:49–50).

#### Theme 1: Parents experienced uncontrolled thoughts regarding their adolescent abusing substances

From the eight interviews that were conducted, it was evidenced that the participants had difficult parental experiences that led to their uncontrolled thoughts. The parents reported that they were fearful that their adolescents would kill them or they would be killed because of their behaviour. This was supported by the following quotation:

‘I fear that when he is under influence of dagga he can kill me. He changed after consuming dagga, I fear him.’ (P4, 52 years old, mother)

Another participant said:

‘I’m not lying that day I was frightened, if he did want to kill me he should have killed me.’ (P7, 48 years, mother)

The participants experienced thoughts of not being good enough parents related to their adolescents abusing substances. Their anxiety could be related to relational conflict, social relationships, lack of cooperation, aggression and experiencing financial strain. This was evidenced by the following:

‘I can’t do anything about it because God gave me this kind of a child.’ (P1, 51 years old, father)

Another participant said:

‘I find it very difficult for me because I’m failing to control him.’ (P2, 49 years old, mother)

The participants experienced wishful thinking regarding their adolescent abusing substances. Parents experienced that the more they pushed their adolescents to work towards a goal, the more the adolescents dug their heels in and resisted them. One of the participants verbalised that she wished her adolescent would die. This was supported by the following quotations:

‘You know what mam those people who beat him at the stadium the time I found him at the hospital, they should have killed him by this time I would’ve forgot.’ (P4, 52 years old, mother)

Another participant also shared:

‘Now is better, I wish that he could stay at the hospital, and never come back.’ (P8, 42 years old, mother)

#### Theme 2: Parents experienced not being able to control their adolescent abusing substances through discipline

Participants experienced their adolescents as misbehaving as displayed by their adolescents abusing substances. Participants shared:

‘When he comes back home late he will be singing, making noise and swearing at people on his way home when I try to talk him he promise to beat me.’ (P1, 51 years old, father)

‘You know what, when he comes back he chase us all. We run and hide at our neighbourhood.’ (P4, 52 years old, mother)

Participants experienced that they were unable to discipline their adolescent because there were no recreational facilities and drugs were easily accessible. The following participant alludes to a parent’s expectation of a child who is obedient within a nurturing environment:

‘I find it very difficult because he is my only son and is not listening to me.’ (P6, 46 years old, mother)

Another participant said:

‘Each and every parent when giving birth to the child he/she expects the child to have good behaviour so that she can take good care of him or herself.’ (P4, 52 years old, mother)

#### Theme 3: Parents experienced negative feelings regarding their adolescent abusing substances

Parents experienced various kinds of emotions because of the events that occurred with their adolescents. Participants confirmed that they struggled to understand what was happening to their adolescents. They did not know how to manage their emotions and blamed themselves, as illustrated by the following quotations:

‘Eish, you know what if it was possible to throw the child away I should have done it so that I can get rest.’ (P3, 48 years old, mother)

‘I think maybe there is something wrong I have done when he grow up.’ (P5, 45 years old, mother)

Participants experienced negative feelings, which depicted feelings of helplessness. This was supported by the following direct quotations:

‘Which school, he dropped out of school at grade 11. Can you imagine a person leaving school at grade 11, he was very intelligent everything is over because of dagga and alcohol.’ (P8, 42 years old, mother)

‘As a parent, I was expecting my child to grow like any other child. Going to school so that he can take care of himself when I’m dead.’ (P1, 51 years old, father)

Parents explained that they were shocked by their adolescent’s behaviour. This is revealed in the following quotation:

‘You know the day I realised that he is smoking dagga, my body and my head changed I was feeling somehow.’ (P7, 48 years old, mother)

She continued:

‘Eish, you know I was not expecting something from his educational side, he is a hard worker and can do something amazing with his hands.’ (P7, 48 years old, mother)

Parents stated that they invested a lot in their adolescents, and it was shocking to see them throwing away their future.

Some participants verbalised that they looked for emotional escape as they were unable to express their emotions of sadness. This emotional escape resulted in unhealthy coping mechanisms. Participants explained:

‘Aaa, I’m very sad because first of all when I call his father so we can help each other as parents, maybe he can respect his father he don’t respond well.’ (P3, 48 years old, mother)

‘I expected my child to go to school so that he can take care of himself when I’m gone, so he left school this makes me sad.’ (P5, 45 years old, mother)

Finally, these parents experienced shame that resulted from disappointment and loss of respect. This is supported by the following direct quotations:

‘My neighbours are blaming me that I’m not giving my children rules.’ (P1, 51 years old, father)

Another participant demonstrated this by saying:

‘He took everything he comes across inside the house and sells it for drugs. The TV that we were having we don’t know where it went. He also sold my utensils.’ (P4, 52 years old, mother)

#### Theme 4: Parents experienced negative consequences regarding their adolescent abusing substances

It was clear from the primary study that negative consequences were the effects, results or outcomes from adolescents abusing substances. The participants noted that their health was compromised. Parents with an adolescent abusing substances also mentioned that they were dealing with stress and anxiety caused by the behaviour of their adolescents. Some even had to rely on chronic medication.

Participants thus experienced negative consequences for themselves. Parents experienced a loss of respect, humiliation and embarrassment to the family and the community. This was supported by the following quotations:

‘I was not taking any treatment but since I heard that he is taking drugs I started taking treatment.’ (P6, 46 years old, mother)

‘I took treatment for a long time, not knowing the problem, I realised that it was him who was causing me stress.’ (P5, 45 years old, mother)

‘I did not expect him to drink alcohol and smoke dagga this way, eeee.’ (P6, 46 years old, mother)

Parents experienced negative consequences of impaired family dynamics and impaired family relationships. One of the participants mentioned that the adolescent was very dangerous whilst abusing substances, and the family members were afraid of him. She said:

‘We use to fight each other in this house because of his behaviour.’ (P4, 52 years old, mother)

Other participants expressed:

‘Now we are teaming up as a family and fight against him.’ (P2, 59 years old, mother)

‘When he comes back home he started arguing with us, he found the knife in the kitchen and he wanted to stab his brother. My children are not safe.’ (P7, 48 years old, mother)

Parents experienced negative consequences from the community. This was supported by the following:

‘As a leader in the community people expect my child to be highly disciplined.’ (P1, 51 years old, father)

Another participant revealed:

‘The stigma is affecting the whole family because of this one child who is abusing substances.’ (P6, 42 years old, mother)

Another participant said:

‘My life has changed here in the community there is this saying that says charity begins at home I just wish that my boy one day could quit the drugs.’ (P2, 49 years old, mother)

## Discussion of the findings

The objective of this study was to explore and describe the experiences of parents with an adolescent abusing substances admitted to a mental institution in Giyani. In this study, the parents with an adolescent abusing substances experienced uncontrolled thoughts regarding their adolescent abusing substances. They also experienced not being able to control their adolescent abusing substances through discipline, as well as negative feelings regarding their adolescent abusing substances. The participants also expressed experiencing negative consequences regarding their adolescent abusing substances.

Uncontrolled thoughts are unwanted thoughts or images that a person finds distressing and/or disturbing. Uncontrolled thoughts, in the context of this study, included unwanted thoughts that were experienced by parents that caused them great distress and seemed to be about their adolescents who were abusing substances.

Parents wanted to talk about intrusive thoughts in terms of how they could arise, the impact they had on them and what they could do to stop them. This fear pervaded their thoughts. It is alluded by Orfort et al. (2010:4–5) that the person abusing substances can also be aggressive and sometimes verbally and physically abusive to people around them. At times, they break things in the house and threaten those around them, which is what leads to most parents feeling unsafe and fearful. Moreover, Orford et al. (2010:4) continue stating that the strain of living with an adolescent abusing substances includes that the person can be aggressive; he or she always seems to be fighting. This often causes the family to feel unsettled and anxious when they are around the adolescents. Parents are also dealing with challenges of ensuring their own safety and that of other members of their families. Hoeck and Van Hal (2012:6) claim that parents worry about the lifestyle of their adolescents who are abusing substances, and the company they keep as they do not know the influence they are getting from these peers also supports this.

The parents shared their feelings of guilt about failing the adolescent, and they believed that they were responsible for their adolescent’s behaviour. They also said that they were perhaps absent when their adolescents needed them most. Self-blame thus came out very strongly as parents experienced guilt and anger at themselves about their adolescents. They thought that neglect might be what led to the adolescent’s abuse of substances. Parents were also emotionally drained as they were obligated to rescue their adolescents in one way or another, and they continued to blame themselves, saying that they should have been better parents to the adolescents (Kirst-Ashman 2015:469). Participants shared that they were responsible for the way their adolescents turned out. They blamed themselves for the lifestyle and behaviour of their adolescents. The studies by Barlow (2010:131) and Choate (2015:468) confirm that some parents blame themselves when their adolescents abuse substances, believing that they failed in their role to guide their adolescents as parents. Some felt ashamed, angry and guilty about their adolescents abusing substances.

The participants who were interviewed articulated that seeing their adolescents destroying their future by dropping out of school caused them stress and really affected them badly. They had dreams of seeing their adolescents with a brighter future. Some parents said that they wished their adolescents did not come back from the psychiatric hospital because their lives were safe, and there was peace in the family when they were absent. Choate (2015:470) corroborates that parents experience that they still love their adolescents, but their experiences make it difficult to live with them; hence, they wish they could die or not be discharged from the hospital.

Parents experienced not being able to control their adolescent abusing substances through discipline. Parents experienced a sense of worthlessness and powerlessness in terms of controlling their adolescent abusing substances. Discipline with love and respect in the family is crucial for any individual’s upbringing.

Parents were miserable because of the behaviour displayed by their adolescents abusing substances. Parents were shocked when their adolescent’s behaviour changed towards them. Some of the parents verbalised how their adolescents became moody and aggressive when they talked to them. According to the study conducted by Dreyer (2012:21), some parents justified their adolescents’ behaviour because they felt that they neglected them. Their adolescents were thus exhibiting odd behaviour, and parents were justifying their adolescents’ delinquent behaviour. It is evident that the participants were afraid to confront the deviant behaviour, thus hiding to protect themselves.

Parents discussed their concern about the bad behaviour of their adolescents, in particular the way they are talking to them. Adolescents abusing substances were not listening to their parents when they told them to do something good. The participants experienced an inability to discipline their adolescents abusing substances. Parents’ roles in the family environment include preparing children for adulthood through rules and discipline (Hoskins 2014). The Centre for Suicide Research and Prevention U of HK (2011:230) concurs that discipline is the universal prevention strategy within the family that may be employed to reduce incidences and onset of delinquency, including substance abuse in early childhood and adolescence.

Parents experienced negative feelings regarding their adolescent abusing substances. Parents experienced negative emotions from time to time, as they were dealing with their adolescents who are abusing substances. Participants expressed sadness and disappointment as they had higher hopes for their children. Parents wanted to see their adolescents being successful in life and able to take care of themselves when they were dead. Parents were unable to transform these negative emotions into positive emotions and behaviour, as their adolescents were not listening to them. Parents also shared their frustration on how treatment had failed them after all the support they gave their adolescents. The study conducted by Hoeck and Van Hal (2012:9) confirms that parents experience helplessness, shock, sadness and shame because of their adolescents’ habits and problems, and in trying to find help for them. They experienced overwhelming emotions and never self-regulated their emotions as they were unable to tolerate any distress (Svoboda 2019:80). Masombuka (2013:81) confirms that adolescents abusing substances sell appliances and various other things from their homes to get money to buy the substances of their choice. This made parents feel helpless at not knowing what to do about their adolescents.

Orfort et al. (2010:5) confirm that family members or parents feel disappointed that the relationship to which they had devoted their lives, as well as the dreams and hopes they had for their adolescents, had been shattered.

Waini (2015:85) suggests that parents have disagreements on how to deal with the adolescent’s addiction. Moreover, according to Orford et al. (2010:4), the strain of living with an adolescent abusing substances includes that person being aggressive or always fighting with family members for money, which might cause family members to feel unsettled and anxious when they are around the adolescent.

Dreyer (2012:03) states that most adolescents who are abusing substances are not doing well at school as their brain functioning might be affected by their lifestyle. The parents experienced shock, sadness and shame because of the behaviour of their adolescents. In addition, Orfort et al. (2010:4) assert that a family who has an adolescent abusing substances can become fractured and can start to disagree over various issues.

Parents experience negative consequences regarding their adolescent abusing substances. Hoeck and Van Hal (2012:1) and Smith and Estefan (2014:11) state that having an adolescent who is dependent on substances can bring about challenges and stress on physical health and well-being of the parents, leaving them overwhelmed. Howard et al. (2010:473) state that the individual abusing substances runs the risk of compromising and exhausting the relationships in the family setting, which lead to family systems being strained. Hoeck and Van Hal (2012:1) also confirm that substance abuse in the family affects the social life of family members and the way they relate to others. Parents moreover experienced negative consequences from the community. Parents understood that the community was tired of the adolescents’ behaviour. Parents shared that the community members were treating them as if they were supporting the behaviour of the adolescents; they did not realise that they were also challenged by this behaviour. Parents shared that the community perceived them as irresponsible parents because their adolescents were abusing substances. However, the parents needed the community to treat them with respect and stop judging them.

Participants demonstrated that they wanted progress in their adolescents’ lives. In the study by Masombuka (2013:100), it was found that despite the emotional challenges in which parents find themselves, they still hope that their adolescents will change and respect them.

## Recommendations

It is evidenced from this research that parents with an adolescent abusing substances admitted to a mental health institution in Giyani face a number of challenges in terms of promoting, maintaining and restoring their mental health. The research was conducted in a rural area with parents to gain a richer understanding of the phenomenon under study. There is a need for further exploration regarding how psychiatric nurses can support parents of adolescents abusing substances in other areas. There is also a need for psychiatric nurses to educate communities about substance abuse amongst the adolescents. School health programmes focussing on substance abuse would be beneficial. Further research studies can also be conducted in other villages to gain a greater understanding of those parents’ experiences with an adolescent abusing substances. It is necessary to manage the mental health of the parents holistically as human beings with mind, body and spirit, who also interact with their external environment physically, mentally, socially and spiritually (University of Johannesburg 2017:4). The researcher recommends that a model should be developed to facilitate the mental health of parents with an adolescent abusing substances admitted to a mental health institution in Giyani.

## Limitations of the study

This research had limitations, including the difficulty experienced in getting willing participants as some feared to verbalise their experiences. The researcher faced constraints regarding the scheduling of appointments with the participants. Participants had busy schedules at their work and home environments. Another limitation that the researcher noted was anxiety; participants were anxious initially but relaxed as the interviews proceeded. The audio recording also added to the participants’ anxiety during interviews. The limitation of unavailability of sufficient time was addressed by means of prolonged engagement to accommodate the participants’ busy schedules. The study was undertaken at the homes of participants, and therefore findings are not representative of all parents with an adolescent abusing substances in Giyani. The author who initially conducted the research on which this article is based also addressed this limitation by indicating that the results are based on the context of this study.

## Conclusion

The findings showed that parents with an adolescent abusing substances faced a number of challenges, as evident from their shared experiences. The results displayed that there was poor social support for these individuals; however, there was no doubt that social support is highly valued by the parents. Participants displayed signs of stress and strain and were employing various coping strategies to try to respond to their situation. There was strong evidence of emotionally burdened experiences by the parents because of their adolescent abusing substance. This emphasises the need for further research to be conducted in developing a model for the facilitation of their mental health. It is evident from their nature of challenges and difficulties experienced that these parents need external interventions to facilitate their mental health.
